# Outstanding animal studies in allergy I. From asthma to food allergy and anaphylaxis

**DOI:** 10.1097/ACI.0000000000000363

**Published:** 2017-04-25

**Authors:** Erika Jensen-Jarolim, Isabella Pali-Schöll, Franziska Roth-Walter

**Affiliations:** a Institute of Pathophysiology and Allergy Research, Center of Pathophysiology, Infectiology and Immunology, Medical University of Vienna; b The Interuniversity Messerli Research Institute, University of Veterinary Medicine Vienna, Medical University Vienna, University of Vienna, Vienna, Austria

**Keywords:** anaphylaxis, asthma, FcgammaR, food allergy, IgE, mouse model, rhinitis

## Abstract

**Purpose of review:**

Animal models published within the past 18 months on asthma, food allergy and anaphylaxis, all conditions of rising public health concern, were reviewed.

**Recent findings:**

While domestic animals spontaneously develop asthma, food allergy and anaphylaxis, in animal models, divergent sensitization and challenge routes, dosages, intervals and antigens are used to induce asthmatic, food allergic or anaphylactic phenotypes. This must be considered in the interpretation of results. Instead of model antigens, gradually relevant allergens such as house dust mite in asthma, and food allergens like peanut, apple and peach in food allergy research were used. Novel engineered mouse models such as a mouse with a T-cell receptor for house dust mite allergen Der p 1, or with transgenic human *hFcγR* genes, facilitated the investigation of single molecules of interest. Whole-body plethysmography has become a state-of-the-art in-vivo readout in asthma research. In food allergy and anaphylaxis research, novel techniques were developed allowing real-time monitoring of in-vivo effects following allergen challenge. Networks to share tissues were established as an effort to reduce animal experiments in allergy which cannot be replaced by in-vitro measures.

**Summary:**

Natural and artificial animal models were used to explore the pathophysiology of asthma, food allergy and anaphylaxis and to improve prophylactic and therapeutic measures. Especially the novel mouse models mimicking molecular aspects of the complex immune network in asthma, food allergy and anaphylaxis will facilitate proof-of-concept studies under controlled conditions.

## INTRODUCTION

Animal models have significantly contributed to the understanding of the three allergy problems with the most destructive outcomes being asthma, food allergy and anaphylaxis. Although mice are the most commonly used species, the immunological limitations of this model have been increasingly realized, prompting for instance the generation of transgenic mice expressing IgE-receptors on cells that are relevant in human allergy, such as dendritic cells or eosinophils. Surprisingly, current studies with such models even suggested an anti-inflammatory role of IgE/FcεRI signals [1], reviewed in [2]. The situation in mice is also complicated by the fact that mouse FcγRIV acts as IgE-receptor similar to human FcεRI and contributes in IgE-induced lung inflammation [3]. More rarely, other allergy models than mice were used, for instance rats and guinea pigs [4,5], or piglets [6], especially for answering pharmacologic questions. In the investigated time period of this review (18 months back), no research articles involving monkey or nonhuman primates were published.

As companion animals may spontaneously develop skin (dogs, cats and horses), respiratory (cats and horses) and food allergies (dogs, cats, horses) like human patients, their inclusion as animal model ‘patients’ is increasingly encouraged [7]. Although in these animals little is known about the eliciting specific allergen molecules [8,9], the immune mechanisms relevant for allergies in dog, cat and horses are more similar to humans than any rodent models. These facts prompted experimental approaches including models for food sensitization [10] or for sublingual allergen-specific immunotherapy [11], up to clinical comparative investigations in animal patients, which are very similar to humans [12,13]. Technically, however, all investigations in animals are regulated by the animal experimentation directives [14]. This may prevent animal owners from participation in trials.

Therefore, and for practical reasons, most current work on animal models discussed below derives from murine studies and allows comparisons with previous mouse murine studies, but there are continuous efforts to advance these artificial models in terms of immune parameters and technical readouts.

### Asthma models: using antigens or allergens?

Asthma in humans develops due to chronic inflammation of the airways, in contrast to murine asthma models that only develop acute airway inflammation [15]. Nevertheless, most of our knowledge about the pathogenesis relies heavily on available mouse models.

Ovalbumin from egg is often used as a model antigen, although it is not a respiratory allergen for humans. The rationale is that a great number of transgenic models have ovalbumin-specific immune cells, allowing elegant and precise studies of the immunological machinery. It should be noted, however, that also transgenic mice exist which specifically react to more relevant allergens, like the 1-DER mice expressing the T-cell receptor specific for the major house dust mite allergen Der p 1 from *Dermatophagoides pteronyssinus*[16]. Recent reports, all using ovalbumin have shown aggravation of asthma by pentraxin 3 [17], prostaglandin E2 [18], IL-15-deficiency [19] or suppression of asthma by anti-inflammatory protein 2 from hookworms [20^▪▪^], natural killer (NK) receptor 1 [21] and inhibition of hypoxia inducible factor-1α [22]. Sensitization with ovalbumin was usually performed by the intraperitoneal route, which, though valid, hardly represents the natural respiratory sensitization process in humans or animals. Allergen challenge to induce the pulmonary phenotype is conducted intranasally or by aerosolization in nebulization chambers (see below, Table 1). 

**Box 1:**
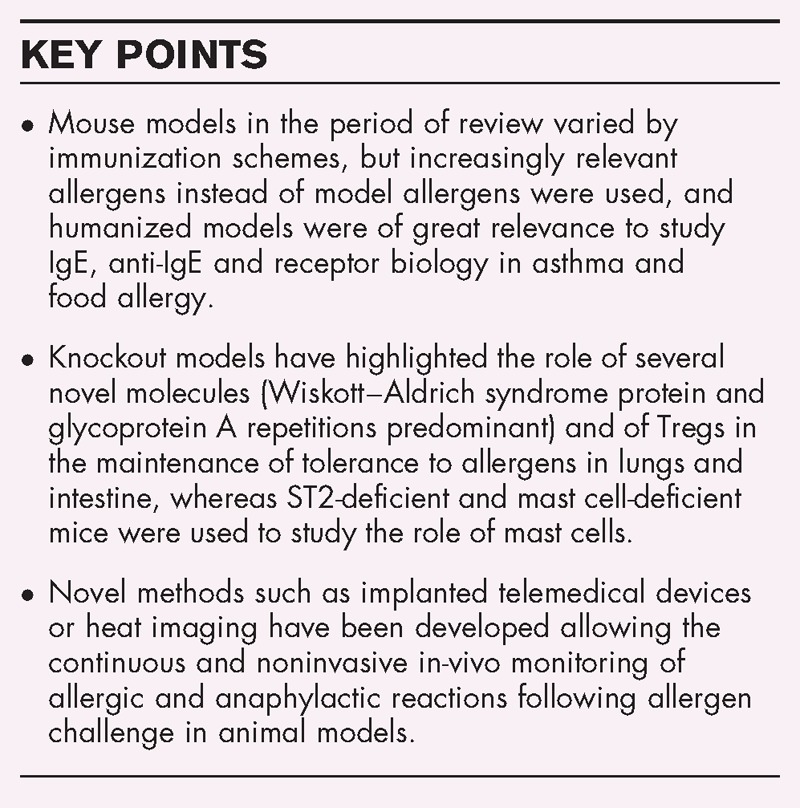
no caption available

An increasing number of current studies tried to mimic the natural sensitization course, mainly using allergen extracts of house dust mites [23–27,28^▪▪^,^29^–^34^], but also from other relevant sources like from the fungus *Alternaria alternata*[35] and cockroaches [36,37].

Also transfer models were employed, in which human blood immune cells stimulated in-vitro with allergens are transferred to immune-deficient mice to mimic human asthma [38].

Considering the increasing number of molecular allergens available, more studies should be done with ‘true’ allergens which, however, will require the creation of new specific reagents and models.

#### Pathophysiology associated with immune changes in asthma models

**FIGURE 1:**
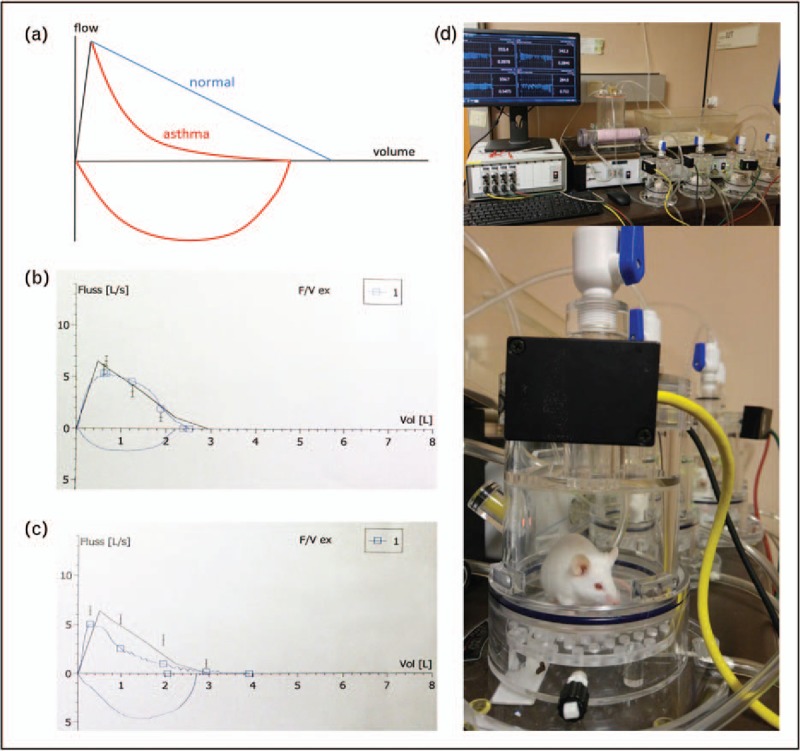
Spirometry: principle and typical test results. (a) Diagram showing the changes in airflow in healthy (blue) and asthmatic condition (red) (Adapted from [43]; (b) original spirometry test results of a human healthy patient; (c) of a patient suffering from bronchial asthma. Note the reduced airflow (upper blue line) due to bronchoconstriction above the *x*-axis in (c), as compared with normal lung function in (b); *y*-axis: forced expiratory flow (FEF) in l/s; *x*-axis: expired air volume in liters; (d) whole-body plethysmography in mouse studies for asthma research (d1, series of measurements in mice; d2, single tested mouse). The experimental principle is identical to the human diagnostic test. Reproduced with permission from Springer [39].

As a readout for airway hyperreactivity and lung resistance, mice were exposed to increasing concentration of methacholine and monitored either by whole-body plethysmography (Fig. 1, from [39]) [29,34,40] or by surgically implanted means [23,24,27]. Studies investigating the mechanisms at the very start of allergic sensitization demonstrated that B-cells are dispensable for the primary CD4^+^ T-cell response, but crucial for expansion of primed Th2 cells and generation of central memory [28^▪▪^], ILC 2 were not an early source of Th2 cytokines, but contributed to allergic inflammation [26] together with IL-33 derived from monocytes [24], IL-23 secreted by bronchial epithelial cells [32], placenta growth factor [27] and lysophophatidylcholine [36]. Th2 and Th17 inflammatory pathways seemed to be reciprocally regulated in asthma [41]. On the other hand, CpG-oligodeoxynucleotides [37], STAT3 inhibition [42], CB2 receptors on NK cells [31] or secretoglobin superfamily protein SCGB3A2 [33] suppressed allergen-induced asthma in mice. Thus, the investigated factors illustrate that asthma can be counterregulated by reinforcing tolerance or skewing Th2-based asthma towards Th1 or Th17.

#### Models of severe asthma

Asthmatic patients immunologically cluster either in Th2-high, Th17-high or Th2/17 subgroups [41]. Severe asthma patients usually have high IFN-γ and low Th2 and IL-17 responses and do not respond to systemic corticosteroids [23]. Thus, models for severe asthma have been developed, in which cyclic-di-GMP (Guanosinmonophosphat), a second messenger produced by many pathogenic bacteria and being a strong Th1-Th17 adjuvant, was coapplied with the allergen extract during sensitization [23]. Similarly, severe asthma was also induced by infecting mice during the sensitization phase with influenza virus [30].

### Food allergy models

#### Novel models: humanized and knockout

As a novel food allergy model, NOD-scidγc^−/−^ mice with a human membrane-bound stem cell factor transgene were engrafted with human cord-blood derived CD34^+^ hematopoietic stem cells (HSCs) [44^▪▪^]. When peanut butter was given intragastrically, HSC-engrafted mice formed peanut-specific serum-IgE, systemic anaphylaxis (body temperature drop and human serum tryptase) and human IgE^+^ mast cells were observed in the small intestine, the spleen and the skin.

Using this model, CD4^+^CD25^+^ splenic T cells stimulated with or without allergen and/or recombinant glycoprotein A repetitions predominant (GARP) were transferred into the mice [45]. After allergen challenge, mini-endoscopy revealed that Tregs were responsible for inhibition of human allergen-specific IgE and allergen-specific gut inflammation. This inhibitory effect of Tregs was abolished when Treg-inducing protein GARP was blocked.

In another study, the phenomenon why Wiskott–Aldrich syndrome (WAS) patients develop higher rates of food allergy was investigated in a WAS protein-deficient mouse model. The results revealed that WAS protein expressed in Foxp3^+^ T cells has a critical function in the control of immune tolerance to chow antigens, such as soy and wheat, and thus maintenance of intestinal tolerance [46]. In summary, novel humanized mouse models confirmed the impact of Foxp3^+^ Tregs in tolerance induction to food.

#### Other animal models of food allergy

In addition to mouse and rat models, all relying on diverse sensitization methods, also pigs, dogs or sheep are used in food allergy research (reviewed by [47]). Epicutaneous sensitization was established in Beagles, where peanut paste was applied to normal and atopic dogs (each group *n* = 5), resulting in serum-IgE in all atopic vs. two control dogs [10]. After oral challenge, pruritic dermatitis, clinical symptoms scores, eosinophilic dermatitis and IgE-positive cells in skin were significantly higher in atopic dogs. This model is useful to mimic food-aggravated atopic dermatitis rather than gastrointestinal symptoms.

#### Microbial compounds, RNA and vitamins

To study the influence of microbial products, Li *et al.*[48] stimulated B cells with recombinant bacterial flagellin together with IL-4 as Th2-cytokine. In-vitro, flagellin suppressed expression of B-cell lymphoma-6 (Bcl6), a transcription factor repressing IgE-switch in B cells. Exposure to flagellin together with IL-4 induced IgE, while flagellin or IL-4 alone did not. In a wild-type mouse model, ovalbumin induced higher specific IgE, mast cells in intestinal mucosa and anaphylactic temperature drop when coapplied with flagellin. The same protocol in flagellin-receptor TLR-5^−/−^ animals prevented these symptoms.

Micro-RNA can also regulate immune responses. B cells of ovalbumin-sensitized mice expressed high levels of the micro RNA miR-19a in the intestinal mucosa, in parallel with low levels of IL-10 [49]. Accordingly, addition of IL-4 increased the expression of miR-19a and suppressed IL-10 also in-vitro.

The physiologically observed neonatal inefficiency to induce oral tolerance to ovalbumin was studied in the context with vitamin A [50]. In neonatal animals, antigen transfer was markedly increased through the gut barrier, and only from the 3rd week of life on, animals were able to establish oral tolerance. The newborn mice showed a specific deficiency of retinaldehyde dehydrogenase expression in CD103^+^ dendritic cells (DC) of mesenteric lymph nodes associated with a reduced ability for antigen-specific T-cell activation, with retinol serum levels three times lower than in adults. When vitamin A was supplemented, oral tolerance could be induced right from birth. Therefore, bacterial compounds such as flagellin and micro-RNAs may potentiate food allergy, while vitamin A seems important for tolerance induction, at least in young age.

#### Novel approaches to treat food allergies

Oral treatment of peanut-allergic C3H/HeJ mice with CpG/peanut-poly(lactic-co-glycolic acid) nanoparticles as novel carrier system protected them from anaphylaxis, decreased peanut-specific IgE/IgG1 and Th2 cytokines [51], in accordance with previous own studies [52].

Antihuman IgE antibodies were used in a novel humanized murine model of peanut allergy: PBMC from human peanut-allergic donors were transferred into NOD-*scid* IL2Rgamma^null^ mice, followed by an intraperitoneal challenge with peanut extract [53]. As therapy, an adeno-associated viral vector coding for a full-length, high-affinity, anti-human IgE antibody derived from the Fab-fragment of Omalizumab was applied once i.v. and significantly reduced specific and total serum IgE, free IgE, plasma histamine, clinical anaphylaxis and prolonged survival times.

Low-dose human recombinant IL-2 as survival factor for Tregs was injected in an ovalbumin- or peanut-allergic mouse model [54]. It prevented food allergy symptoms, anaphylactic body temperature drop, decreased serum mast cell protease-1 (MCP-1) and IL-5 in mesenteric lymph nodes and increased ovalbumin-specific IFN-γ production in mesenteric lymph nodes and Peyer's patches. Reduction of symptom scores was only effective after about 2 weeks. Anti-CD25 mAb treatment proved the protective role of Tregs in this model.

Mucosal mast cells (MMCs) are positively correlated with diarrhea and anaphylactic symptoms in murine food allergy. In ovalbumin-sensitized mice [55], pharmacologic inhibition of Notch-signaling by a γ-secretase-inhibitor suppressed food antigen-induced mast cell hyperplasia in the intestinal tract. Only 30% of treated vs. 89% of control animals showed allergic diarrhea.

A recombinant hypoallergenic mutant of the major carp allergen Cyp c 1 (mCyp c 1) was used to produce antisera in rabbits or mice [56]. When mice were sensitized to Cyp c 1 or carp-extract and received the antisera, allergic symptoms after oral challenge with Cyp c 1 and carp extract were markedly reduced. The induced specific IgG1 also reduced Cyp c 1-specific degranulation of IgE-loaded rat basophilic leukemia cells by 95%.

Plasmid DNA encoding the peanut-protein (p) Ara h 2 or pAra h 2 mixed with poly-l-lysine were injected intradermally either before or after sensitization with Ara h 2 [57]. The treatment containing poly-l-lysine was more effective in preventing specific serum antibodies and temperature drop upon challenge when applied before sensitization, inducing CD207^+^ DCs in draining lymph nodes of skin, CD25^+^Foxp3^+^ Treg cells in lymph nodes and spleen, as well as IFN-γ and IL-10 in splenocytes.

The natural molecule diosgenin from Chinese yam was administered daily to ovalbumin-sensitized BALB/c mice concomitantly with repeated ovalbumin challenges and increased the number of Th1-like Treg cells (Foxp3^+^, but coexpressing Th1 markers like CCR5, CXCR3, IFN-γ and T-bet) in the intestine, as well as in Peyer's patches [58].

Another natural molecule, baicalein (5,6,7-trihydroxyflavone) from Baikal skullcap (*Scutellaria baicalensis*) used in Traditional Chinese Medicine, supported integrity of intestinal tight junctions and prevented uptake of antigens and subsequent food allergy [59]. In an ovalbumin-food allergy mouse model, baicalein – an agonist of the aryl hydrocarbon receptor – reduced allergic symptoms (diarrhea, anaphylactic response, body temperature drop, serum IgE and effector T cells), whereas TGF-β was upregulated. As safety in food allergy therapy is a major concern, strategies to reduce the allergenicity of the therapeutic allergen by encapsulation or application of hypoallergens – besides inhibition of IgE pathways – may be most promising.

### Anaphylaxis models

#### Allergens and routes investigated

Anaphylaxis models are mostly used in food allergy research with paradigmatic allergens like ovalbumin and intraperitoneal injections followed by oral challenges. A recent study used peach allergen Pru p 3 with lipopolysaccharide as adjuvant for intranasal sensitization, and anaphylaxis could be elicited by intraperitoneal challenge [60]. The authors suggested this model to be suitable for testing immunotherapeutic strategies. In another model hazelnut was applied orally and intraperitoneally. Usage of the Th2-adjuvant aluminium hydroxide (alum) led to stronger anaphylactic responses than using Staphylococcal enterotoxin B as adjuvant, which could in fact be therapeutically addressed by proteasome inhibitor bortezomib, reducing IgE and the B-cellular arm [61]. The question of the participation of IgG in the observed reactions was not addressed in this model.

PR-10 family members Bet v 1 from birch and Mal d 1 from apple induce clinically cross-reactive IgE in humans. To address whether allergen-specific immunotherapy (AIT) may affect cross-sensitization, mice were serially sensitized to both molecules with alum intraperitoneally up to a level when Mal d 1 elicited anaphylaxis. When mice then were subcutaneously treated with birch pollen extract adsorbed to alum, like in some human AIT formulations, this reduced the anaphylactic response [62]. Instead of causal treatment, histamine-1-receptor antagonist loratadine and histamine-4-receptor antagonist JNJ7777120 protected peanut-sensitized mice from intestinal anaphylaxis, potentially via preventing intestinal DC accumulation and antigen presentation to T cells [63]. Along this line, another study suggested that histamine-4-receptor antagonist JNJ28307474 should be applied already during the sensitization process to prevent allergy [64].

Taken together, only one of the above studies made efforts to truly mimic the oral sensitization route or used oral challenge with a food allergen, and all used Th2 adjuvants to induce anaphylaxis.

#### Addressing IgG mechanisms of anaphylaxis

To exclude any biases in terms of induced antibody isotypes in anaphylaxis models (see below), an adjuvant-free sensitization protocol was developed using the intraperitoneal route and ovalbumin [65^▪^]. Knockout models indicated that the anaphylactic drop of body temperature depended on mouse FcεRI, FcγRIII and γ-chain FcRγ, whereas FcγRIV related to inflammation only [65^▪^]. In this model, macrophages and neutrophils turned out to be less important in anaphylaxis than mast cells.

IgG-mediated anaphylaxis has recently become an emerging concern in humans too and can be mimicked by mouse models [66], in which besides IgE, also IgG (via FcγRIV) may act anaphylactogenic [3]. Different mouse IgG subclasses, however, have different effects depending on the used IgG receptor. The tested mouse IgG1 turned out to be exclusively activating and anaphylactogenic when triggered by the model allergen trinitrophenyl-bovine serum albumin, whereas IgG2a and IgG2b also bound to the inhibitory FcγRIIb receptor. This suggested that the sum of these reactions may decide on the anaphylaxis [67^▪^]. Efforts have been done to translate these findings into the human setting, in which four IgG subclasses complicate the events. A transgenic mouse was generated with human *hFcγR* genes (encompassing activating hFcγRIIA, hFcγRIIIA, hFcγRIIIB and inhibitory hFcγRIIB) being inserted into the corresponding mouse locus. Experiments with FcγR locus-switched mice injected with human heat-aggregated intravenous immunoglobulins suggested that despite the presence of inhibitory FcγRIIb, the activating receptors dominated the outcome of the anaphylaxis experiment [68^▪▪^].

#### Percutaneous sensitization and anaphylaxis

Also percutaneous exposure to food, especially in atopic dermatitis, may lead to sensitization, priming or boosting of existing allergy. The role of IL-33 binding to the IL-33 receptor ST2 (expressed in human and mouse skin) to trigger anaphylactogenic Th2 responses was investigated. Wild-type, ST2-deficient and mast cell-deficient KitW-sh/W-sh mice were percutaneously sensitized to ovalbumin and orally challenged [69]. Although food-allergic wild-type mice experienced anaphylaxis, it was attenuated in ST2-deficient and in ST2-blocked animals, and abrogated in mast cell-deficient KitW-sh/W-sh mice. The study highlighted the importance of IL-33 and mast cells to trigger anaphylaxis and might lead to novel anti-IL-33 therapeutics in humans [69]. Interestingly platelets, which participate in the anaphylactic reaction, have proven a significant source of IL-33 in humans as well as in an airway eosinophilia model in mice [70].

#### Novel readout methods

Following the 3R rules (replacement, reduction and refinement of animal experiments), efforts are done to minimize the number of animals used in the experiments, for instance by sharing tissues in networks as SEARCH [71]. However, in-vivo anaphylaxis models are needed in allergy research and for safety monitoring in vaccine development. Following single animals continuously over time during a reaction reduces the number of animals needed during an experiment.

**FIGURE 2:**
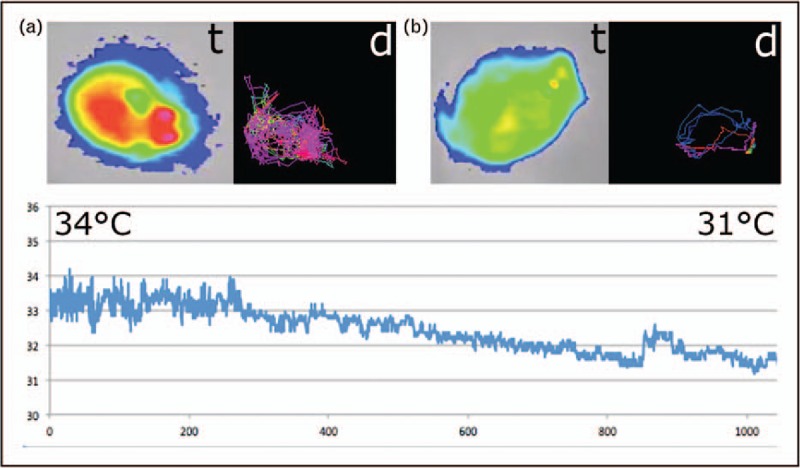
(a) Anaphylaxis imaging in mice with a heat-sensitive camera. The surface body temperature (*t*), in a healthy mouse around 34 °C, is shown in false colors: hottest point in red (usually the head) to > yellow > green > blue (coldest areas). Simultaneously, horizontal mouse movements can be electronically recorded and the running distance (*d*) calculated. (b) Anaphylaxis is associated with a drop of body temperature. Here, values around 31 °C are shown (*t*), associated with impairment in physical activity and shortest distances moved (*d*) [73]. The curve below shows the continuous evaluation of the surface body temperature drop (*y*-axis) recorded using 1000 frames per second, that is over approximately 4.1 min of an anaphylactic reaction (*x*-axis). The vertical movements evaluation is not depicted.

The major anaphylaxis readouts in mouse models are drop of body temperature, reduction of physical mobility, eventually diarrhea and the elevation of MCP-1 levels released from anaphylactic mast cells. Telemedical devices can be surgically implanted into mice that allow the live reading of temperature, but this invasive method requires an experienced hand. Alternative methods based on imaging can only be done after isoflurane anesthesia and are usually not suitable for monitoring animals in motion [72]. A novel device and software has been developed that allows the noninvasive monitoring of surface body temperature and thereby also horizontal and vertical movements by imaging heat pictures (Fig. 2) [73]. Proof-of-concept studies were successful using casein, peanut and ovalbumin as food allergens for sensitization of BALB/c mice.

## CONCLUSION

Summarized, a plethora of current murine asthma models helped us to an improved understanding of the pathophysiology of human asthma, food allergy and anaphylaxis. The routes, dosages, intervals and antigens used for sensitization, or used for challenges to induce an asthmatic (Table 1) or food allergic phenotype (Table 2), are, however, very diverse and should be considered in the interpretation. Increasingly, more relevant allergens such as house dust mite in asthma, and as peanut, apple or peach in food allergy research are used. In-vivo readouts are important to assess the ‘clinical’ relevance. This is done by whole-body plethysmography in asthma research (Fig. 1) [39]. In food allergy and anaphylaxis research, novel techniques were developed allowing the in-vivo monitoring of effects by allergen challenges, and, in accordance with the 3R principle, networks are formed to share tissues [70]. Future research directions may address the development of novel engineered mouse models facilitating the investigation of single-target molecules, whereas domestic animals represent natural animal models, spontaneously developing asthma, food allergy and anaphylaxis with immune mechanisms more similar to human allergy.

## Acknowledgements

No funding was obtained from National Institutes of Health (NIH), Wellcome Trust and Howard Hughes Medical Institute (HHMI).

### Financial support and sponsorship

The work to this review was supported by grant SFB F4606-B28 of the Austrian Science Fund FWF to E.J.-J.

### Conflicts of interest

E.J.-J is inventor of the Imaging method for non-invasive temperature and physical activity measurement in small animals EP 13158620.8-1657 and shareholder of Biomedical International R+D GmbH., Vienna, Austria. The other authors declare no conflicts of interest.

## REFERENCES AND RECOMMENDED READING

Papers of particular interest, published within the annual period of review, have been highlighted as:

▪ of special interest
▪▪ of outstanding interest

## Figures and Tables

**Table 1:** Sensitization and challenge schemes used in murine asthma models

Allergen	Sensitization	Challenge	AHR	Reference
*Alternaria alternata* extract	iTreg cells transferred i.v. into Rag2^−/−^ mice followed by i.n. with IL-33 or 25 μg *Alternaria alternata*	n/a	i.t.	[35]
Amb a 11	2× 10 μg Amb an 11 i.p.	4× 10 mg Amb a 1 + 100 μg LPS aerosol, 20 min	WBP	[40]
Birch allergen	1× 5 Mio PBMCs + 20 μg birch allergen + 10 ng IL4 i.p. 1× 20 μg birch allergen + 10 ng IL4	3× 20 μg birch allergen	i.t.	[38]
CE	3× 10 μg CE i.p.	3× 10 μg CE i.n.	WBP	[36]
CE	6× 120 μg CE + 3 μg CpG-ODN i.n.	n/a	WBP	[37]
HDM	1× 200 μg, 2× 100 μg i.n.	4 cycles: 2× 50 μg HDM i.n.	WBP	[41]
HDM	Severe asthma:3× 25 μg HDM + 7 μg c-di-GMP	3 cycles with: 1× 25 μg HDM + 5 μg cyclic-di GMP and 2× 25 μg HDM i.n.	i.t.	[23]
HDM	3× 25 μg HDM i.n.	3× 5 μg HDM i.n.	i.t.	[24]
HDM	3× 25 μg HDM i.n.	n/a	WBP	[25]
HDM	1× 1 μg HDM i.n.	5× 10 μg HDM i.n. or 1× 100 μg HDM i.n.	n/a	[26]
HDM	2× 10 μg HDM i.p.	100 μg i.t. on day 14 + 19	i.t.	[27]
HDM	1× 1 μg crude HDM i.t.	2× 10 μg HDM i.n.	j.v., WBP	[28^▪▪^]
HDM	5 cycles: 5× 25 μg HDM i.n.	n/a	WBP	[29]
HDM	5 cycles: 3× 25 μg HDM i.n. 1× influenza virus ×31 i.n.	n/a	WBP	[30]
HDM	1× 100 μg HDM i.n.; 4× 50 μg HDM i.n.	n/a	n/a	[31]
HDM	4× 50 μg HDM i.n.	3× 50 μg HDM i.n.	n/a	[32]
HDM	5 cycles: 5× 25 μg HDM i.n.	n/a	n/a	[33]
HDM	3 cycles: 5× 40 μg HDM i.n. and 1× 320 μg HDM i.p.	n/a	n/a	[42]
HDM + DEP	3× 1 μg HDM + 25 μg DEP i.p.	n/a	WBP	[34]
KLH	3 cycles: 2× 5 μg KLH ± 10 μg acrolein, 2 cycles: 2× 10 μg KLH ± 20 μg acrolein i.n.	2× 10 μg KLH i.n.	WBP	[74]
OVA	2× OVA + Alum i.p.	2× OVA i.n.	i.t.	[17]
OVA	2× 20 μg OVA + Alum i.p.	5× 0.2% OVA aerosol, 20 min	i.t.	[20^▪▪^]
OVA	3× 25 μg OVA + 2 mg Alum i.p.	3 or 7× 1% OVA aerosol, 1 h	n/a	[19]
OVA	2× OVA i.p.	5× OVA i.n.	i.t.	[22]
OVA	2× 0.1 μg OVA + 1 mg Alum i.p.	2× 25 μg OVA i.n.	n/a	[21]
Ova	3× 20 μg Ova with Alum i.p.	3× 1% OVA aerosol, 30 min	i.t.	[18]

AHR, airway hyperreactivity; CE, cockroach extract; DEP, diesel exhaust particles; HDM, house dust mite extract; i.n. intranasal; i.p. intraperitoneal; i.t. intratracheal; i.v.intravenous; j.v. jugular vein; KLH, keyhole limpet hemocyanin; LPS, lipopolysaccharide; n/a, not applicable.; OVA, ovalbumin; PBMC, peripheral blood mononuclear cells; WBP whole-body plethysmography.

**Table 2:** Sensitization, challenge and readout principles in food allergy models

Allergen	Sensitization	Challenge	Readout	Ref.
Chaw components (wheat, soy bean, wheat middlings, yellow corn,fish meal)	*Was*^*−/−*^*, Was*^*−/−*^* Il4*^*−/−*^*, Was*^*−/−*^* Rag2*^*−/−*^ mice: none (spontaneous sensitization)	None (spontaneous sensitization)orstarving for 3 h then fed 12.5 mg soy protein in 300 μl PBS	Surface LAMP-1,Intestinalmast cell expansion,Serum levels of mast cell protease 1 (MCPT1),body temperature by transponder	[46]
Cyp c 1;Hypoallergenic mCyp c 1;carp extract	C3H/HeJ mice: 5× i.g. weekly 100 mg rCyp c 1 + 20 mg cholera toxin in 200 ml 0.2-mol bicarbonate buffer (pH 9)20 mg hypoallergenic mCyp c 1 + Alum s.c. 6 times every month;Carp extract–sensitized C3H/HeJ, 600 ml of heat-inactivated mCyp c 1-specific mouse antisera i.p.	i.g. 100 mg rCyp c 1i.g. 10 mg carp extract	Symptom score	[56]
OVA	BALB/c mice: 2× i.p. 50 mg of OVA + Alum + magnesium hydroxide	5× i.g. 50 mg of OVA every other day	Diarrhea,rectal temperature	[55]
OVA	BALB/c mice: gavaged daily with diosgenin	repeatedly i.g.OVA	Foxp3^+^ Treg cells coexpressing Th1-type transcription factors, cytokines, chemokines in intestine,mRNA expression of chemokines corresponding to Th1-like Treg cells	[58]
OVA	C57BL/6 mice or TLR5^−/−^:i.g. 0.1 mg OVA/mouse ± flagellin + cholera toxin (0.02 mg/mouse) weekly for 4 consecutive weeks	gavage-fed OVA (10 mg/mouse in 0.3 ml physiological saline)	Mast cell infiltration in the intestinal mucosa, intestinal CD4+ T-cell proliferation, core temperature, diarrhea	[48]
OVA	BALBc/c mice: 2× i.p. 20 μg OVA + Alum	5× orally 50 mg OVA every 3 days;Baicalein (20 mg/kg) orally administered daily from day 28–40	Diarrhea, anaphylaxis,rectal temperature	[59]
OVA	C57BL/6 mice or miR-17–92^fl/fl^: fed OVA 1 mg/mouse + cholera toxin (20 μg/mouse) weekly for 4 weeks;wild and miR-19a-deficient mice with recombinant IL-4 or/and LPS via i.p. injection daily for 5 days		Mast cell and eosinophil infiltration in intestinal mucosa, allergen-specific CD4+ T cells in the intestine;B cells isolated from intestine analyzed for IL-10 expression	[49]
OVA	BALB/C (AnNR/J) mice: i.p. 2 × 10 mg OVA + Alum	OVA p.o. (20 mg/mouse), 5× within a 10-day period	Rectal body temperature,clinical score (diarrhea severity, appearance of hirsute pelage)	[54]
OVA	OVA (2 mg) i.g. 3×/week for lactating mothers 3 weeks after delivery or during lactation period ± VitA supplemention by maternal vitamin A-enriched diet from 2 days before delivery until the end of first week	6–8-week-old adult mice: allergic airway inflammation to OVA by i.p. 10 mg OVA + Alum on days 0 and 7, day 17–21 mice exposed to OVA (0.5%) aerosols for 20 min in nebulizer	Serum OVA-specific IgE, IgA,eosinophils BAL;lung cell cytokinesecretion;*ex vivo* gut permeability;small intestine histology;serum retinol levels	[50]
Peanut extract	NOD-*scid* IL2Rgamma^null^ mice: transferred i.p. 3 × 10^7^ bloodmononuclear cells from peanut allergic donors + 100 mg crude peanut extract;i.p. 2× 100 mg of crude peanut extract;therapy: AAVrh.10 anti-hIgE (10^11^ genome copies)at week 3, reconstituted with human donor blood mononuclear cells at week 0	i.g. 300 mg crude peanut extract at weeks 5–10	Clinical score,anaphylaxis score,histamine in plasma,PCA	[53]
Peanut butter	huNSG mice: i.g. 22.5 mg (5 mg protein) skippy creamy peanut butter in 250 ml 0.2 mol sodium bicarbonate, pH 8.0, weekly for 8 weeks	Fed 350 mg peanut butter in 0.2 mol sodium bicarbonate	Temperature measurements using s.c. microchip transponders;PN-specific human IgE in serum;tissue mast cell expansion	[44^▪▪^]
Peanut extract,pAra h 2,PLL-pAra h 2	BALB/c mice: i.d.PLL-pAra h 2 or pAra h 2 (25 μg pAra h 2 DNA) 3×, followed i.p. 2.5 μg purified Ara h 2 + Alum	i.p. 5 mg CPE	Rectal temperature	[57]
Peanut extract	i.p. 3× 500 mg peanut extract + Alum at 1 week interval	7× orally peanut extract (15 mg) every 2 days	Body temperature, clinical symptoms:activity/lethargy, diarrhea, death	
Peanut	C3H/HeJ mice: oralpeanut + cholera toxin;therapy: 4× fed CpG/PN-nanoparticles	5× monthly oral peanut	Visualsymptom scores, body temperature	[51]
Different allergens according to allergy of human donors	NOD.CB17-Prkdc^scid^/J γc^−/−^mice i.p. with 1 × 10^7^ PBMCs from donors allergic to different allergens ± Treg cells (ratio of 1 : 10 or 1 : 20) ± respective allergen (20 mg) ± 4 mghuman recombinant GARP	i.p. allergen boost200 ml 0.9% NaCl	High-resolutionvideo endoscopicrectally	[45]
Peanut paste	Atopic beagles:e.c. 2× weekly for 8 weeks under occlusion on the axillae	Day 56: orally with roasted peanut (2 g/kg)day 66 e.c. peanut paste on one pinna under occlusion	Systemic signs (e.g. vomiting, diarrhea, collapse), pruritus, CADESI,skin biopsies	[10]

AAVrh.10anti-hIgE, adeno-associated rh.10 serotype vector coding for a full-length, high-affinity, antihIgE antibody from Fab fragment of anti-hIgE mAb omalizumab; Alum, aluminum hydroxide; e.c., epicutaneous; GARP. glycoprotein A repetitions predominant; huNSG, nonobese diabetic severe combined immunodeficient common gamma chain-deficient stem cell factor; i.d., intradermal; i.g., intragastric; i.p., intraperitoneal; LPS, lipopolysaccharide; OVA, ovalbumin; p.o., per os; pAra h 2, plasmid encoding Ara h 2; PBMC, peripheral blood mononuclear cells; PCA, passive cutaneous anaphylaxis; PN, peanut; PLL-pAra h 2, pAra h 2 pretreated with poly-L-lysine; WASP, Wiskott–Aldrich syndrome protein.
